# Separation of Short Single- and Double-Stranded DNA Based on Their Adsorption Kinetics Difference on Graphene Oxide

**DOI:** 10.3390/nano3020221

**Published:** 2013-04-04

**Authors:** Po-Jung Jimmy Huang, Juewen Liu

**Affiliations:** Department of Chemistry, Waterloo Institute for Nanotechnology, University of Waterloo, Waterloo, Ontario, N2L 3G1, Canada; E-Mail: p8huang@uwaterloo.ca

**Keywords:** graphene, DNA, adsorption, fluorescence, separation, gel electrophoresis

## Abstract

Separation of short single- and double-stranded DNA typically requires gel electrophoresis followed by DNA extraction, which is a time consuming process. Graphene oxide adsorbs single-stranded DNA more quickly than double-stranded ones, allowing for selective removal of the former with a simple mixing and centrifugation operation. The effect of DNA length and salt on adsorption selectivity has been characterized and its application in DNA melting curve measurement has been demonstrated.

## 1. Introduction

DNA hybridization is a fundamental process in biology and biotechnology [1,2,3,4,5,6,7]. A precise control of the hybridization stoichiometry is crucial for the development of many DNA-based assays and for related biophysical studies. A simple hybridization reaction involves only two complementary DNA strands and more complex reactions involve more than one hundred DNAs, such as in the case of DNA origami preparation [8]. For certain applications, it is desirable to achieve complete hybridization, so that no free single-stranded (ss) DNA are present. For example, in the case of studying DNA folding using fluorescence resonance energy transfer (FRET), unhybridized DNA might fold differently to complicate data analysis [9,10,11]. Other examples include the development of catalytic beacons or aptamer beacons where fluorophore and quencher labeled DNAs are hybridized [12,13]. The presence of free ss-DNAs in such systems results in either increased background or suppressed signal.

DNA concentration is usually measured based on light absorption at 260 nm. However, errors associated with such measurements are known to be quite large, making it difficult to determine the exact stoichiometry [14]. In some systems, certain DNA strands are intentionally added in excess to consume other strands. Therefore, the presence of ss-DNA is quite common in DNA-based assays.

Selective removal of ss-DNA is typically achieved using non-denaturing gel electrophoresis. Based on their mobility difference, ss- and ds-DNAs can be separated on gel [10]. The excised product band is crushed and soaked in buffer to extract the purified product. While this protocol is commonly carried out by many laboratories, this is a very time consuming and technically demanding process. Without a fluorophore label or staining, low concentrations of DNA cannot be visually observed within a gel, making it impossible to carry out such a protocol. In addition, components in the gel (e.g., acrylamide) may interfere with the detection, especially in techniques where high DNA purity is required such as in nuclear magnetic resonance (NMR) studies. While commercial kits are available for removing ss-DNA such as PCR primers, they generally require that the ds-DNA product to be longer than 100 base pairs (bp). For most analytical or biophysical studies, however, oligonucleotide lengths are shorter than 100 bp. Therefore, new methods are needed to achieve this goal.

**Figure 1:**
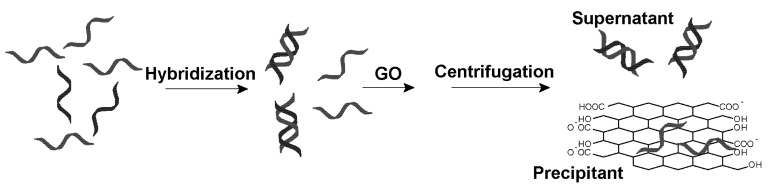
A strategy for graphene oxide (GO)-based selective removal of single-stranded (ss)-DNA. In a mixture of ss- and double stranded (ds)-DNA, GO adsorbs the former more quickly to achieve separation. After a simple centrifugation step, only ds-DNA is left in the supernatant solution.

Graphene is a single layer of graphite with a very large surface area [15,16,17]. To disperse in water, negatively charged graphene oxide (GO) with surface carboxylic acid and hydroxide are often prepared. GO can strongly adsorb ss-DNA through hydrophobic interactions, aromatic stacking and hydrogen bonding [18,19,20,21,22,23,24,25]. For ds-DNA, however, the DNA bases are shielded and only negatively charged phosphate groups are exposed. As a result, ds-DNA adsorption onto GO is possible only in the presence of high salt [26,27]. Given that GO can be easily precipitated via centrifugation, we aim to test the use of GO for selective removing ss-DNA as shown in Figure 1.

## 2. Results and Discussion

**Figure 2:**
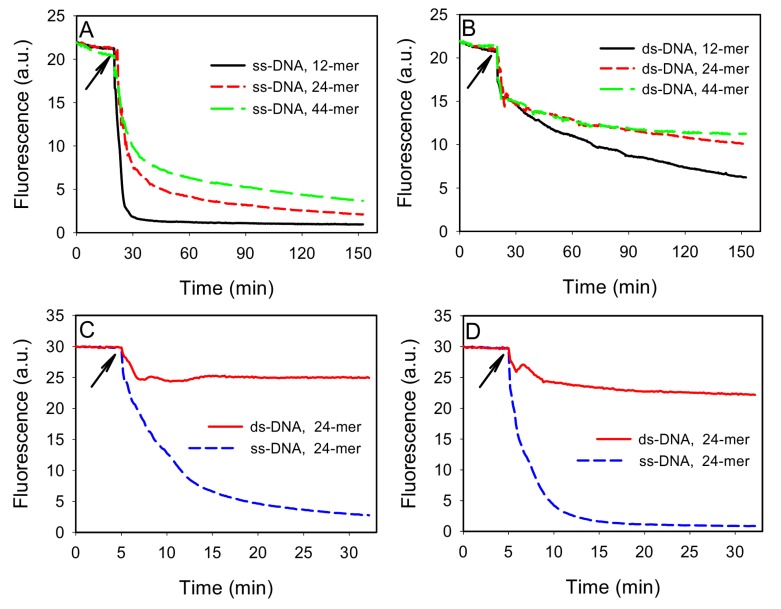
Kinetics of fluorescence change indicative of DNA adsorption by GO for ss-DNA (**A**) or ds-DNA (**B**) of different length. In (**B**), FAM-DNA:cDNA=1:1.5. The buffer contained 100 mM NaCl, 25 mM HEPES, pH 7.6. Comparison of ss- and ds-DNA adsorption kinetics in 100 mM NaCl, 25 mM HEPES with 0 mM (**C**) or 1 mM (**D**) Mg^2+^, where FAM-DNA: cDNA=1:3. The arrows indicate the time point when GO was added.

To test this idea, we first compared the adsorption of ss- and ds-DNA by GO. Three FAM-labeled ss-DNAs of 12, 24, and 44-mer were respectively dissolved in a buffer containing 100 mM NaCl, 25 mM HEPES, pH 7.6. As shown in Figure 2A, addition of 40 μg/mL of GO quickly reduced the fluorescence intensity due to DNA adsorption, since GO is an excellent fluorescence quencher. Shorter DNAs were more quickly adsorbed, consistent with previous reports [20,21]. To prepare ds-DNA, 100 nM of the FAM-labeled DNAs were respectively hybridized with 150 nM of their complementary DNA (cDNA). The cDNAs were used in excess to minimize free FAM-labeled ss-DNAs. As shown in Figure 2B, addition of GO induced a quick fluorescence drop followed by a slow decay kinetics. The initial drop was attributed mainly to light absorption by the added GO. The subsequent slow kinetics was attributed to be the adsorption of ds-DNA. By comparing Figure 2A,B, it can be concluded that the ds-DNA adsorption kinetics was much slower than that for the corresponding ss-DNA, making it possible to separate these two. Interestingly, shorter ds-DNAs still adsorbed more quickly than the longer ones (Figure 2B). This may be explained by that shorter DNAs carried a smaller amount of negative charges and also diffused faster. Thus, they were able to reach the GO surface more quickly. Recent simulation work has shown that ds-DNA is adsorbed either vertically via end base pair stacking on graphene or horizontally by partial opening of the end base pairs [28].

While ss-DNA adsorbed more quickly, ds-DNA adsorption may catch up after a long time. To optimize the adsorption selectivity, we next tuned the relative concentration of DNA and GO, as well as salt. Given that both ss- and ds-DNA can be adsorbed and they compete for surface binding sites, we propose that high selectivity for adsorbing ss-DNA can be achieved by matching the GO adsorption capacity with the amount of ss-DNA. This can generally be realized experimentally since the amount of free ss-DNA can often be estimated. To test this, we used 100 nM of the 24-mer FAM-labeled DNA and increased its cDNA to 300 nM. The kinetics of fluorescence change was then monitored in the presence of 30 μg/mL of GO in 50 μL volume. As shown in Figure 2C, addition of GO resulted in ~20% fluorescence drop. After that, the signal became stable and no ds-DNA adsorption was detected. For comparison, ~90% of the FAM-labeled ss-DNA was adsorbed in ~30 min. Therefore, by filling the GO surface with ss-DNA, subsequent ds-DNA adsorption was inhibited to give a high selectivity. We further optimized the adsorption kinetics by adding divalent metal ions. For example, with an additional 1 mM Mg^2+^, as shown in Figure 2D, close to complete quenching of the ss-DNA fluorescence was achieved in 15 min and the adsorption of ds-DNA remained slow.

**Figure 3:**
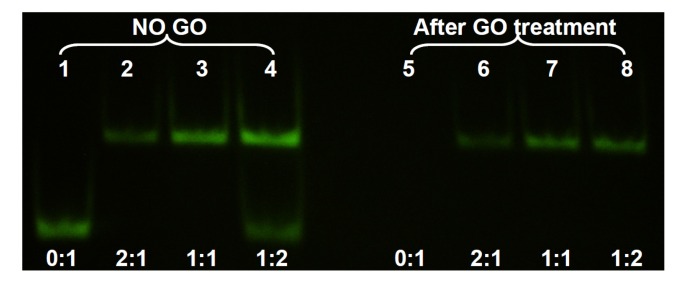
Non-denaturing polyacrylamide gel electrophoresis of the supernatant solution after DNA adsorption by GO and centrifugation. The lower bands are the ss-DNA and the upper bands are the ds-DNA. After GO treatment, only ds-DNA was left in the supernatant.

In the above experiments, adsorption of ss- and ds-DNA was monitored separately. In addition, GO was still present in the reaction mixture after adsorption. For most applications, it is desirable to remove the added GO to eliminate its interference. To test the separation of ds-DNA from adsorbed ss-DNA, we next studied the system using non-denaturing gel electrophoresis. In this experiment, the concentration of the FAM-labeled 24-mer DNA was set at 1, 2, and 4 μM while its non-fluorescent cDNA concentration was fixed at 2 μM. After hybridizing the DNAs, GO was added. For comparison, a set of GO-free samples were also prepared. After 20 min incubation in 100 mM NaCl 25 mM HEPES buffer, the samples were centrifuged and the supernatant was loaded into the gel. As shown in Figure 3, lane 1 was the FAM-labeled ss-DNA to serve as a marker. Lane 2 had 1 μM the FAM-DNA and 2 μM cDNA, producing only the ds-DNA band at a higher position. Addition of GO slightly reduced its intensity and all the FAM-DNA remained in the ds-form (lane 6), consistent with the above fluorometer studies. Lane 3 contained the 1:1 ratio of the two DNAs, both at 2 μM. Therefore, its signal was stronger than that in lane 2. Addition of GO resulted in a similar effect (lane 7). In lane 4, the FAM-DNA was in excess and two bands were observed. Addition of GO removed the ss-DNA in lane 8, where only the ds-DNA band was left. Lane 5 had only the ss-FAM DNA that was completely removed by GO. This experiment confirmed that GO was able to selectively remove ss-DNA in a mixture of ss- and ds-DNA, thus achieving the separation of these two. It is important to estimate the amount of GO needed so that only ss-DNA is removed while most ds-DNA is retained in the solution phase.

**Figure 4:**
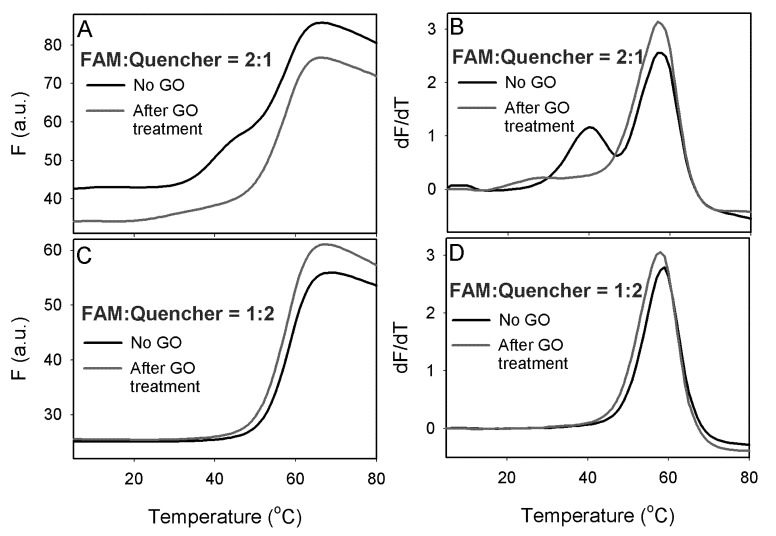
Melting curves (**A**) and its first derivative (**B**) for DNA duplexes made with the FAM-labeled DNA in excess. Melting curves (**C**) and its first derivative (**D**) for DNA duplexes made with the quencher-labeled DNA in excess.

To demonstrate an application of this method, we carried out a DNA melting experiment with a 12-mer duplex. One strand of the duplex contained a FAM fluorophore at the 5'-end and its cDNA was modified with a 3'-end quencher. Melting of this ds-DNA was accompanied with a fluorescence increase. These two DNA strands were mixed at two ratios of 2:1 and 1:2. When the FAM-DNA was in excess, a high background signal was produced as shown in Figure 4A (black curve). In addition, two melting transitions were observed. These two transitions were more obvious by taking the first derivative of the melting curve (Figure 4B). The higher temperature transition was due to the melting of the duplex and the lower transition was attributed to the free FAM-labeled ss-DNA, where the fluorophore might be quenched by certain secondary DNA structures. In this case, the DNA backbone, possibly the guanine bases acted as a quencher [29]. After incubating the DNA samples with 20 μg/mL of GO and centrifugation to precipitate GO, the background fluorescence was significantly reduced and the first melting transition was also eliminated (Figure 4A,B, red curves), suggesting that free FAM-labeled ss-DNA was adsorbed by GO. The height of the second transition and the melting temperature values was not changed by adding GO, confirming that only the ss-DNA was adsorbed. For the sample with a DNA ratio of 1:2, where the quencher labeled DNA was in excess, GO did not affect the melting transition as shown in Figure 4C,D. Therefore, GO selectively adsorbed only the free quencher labeled DNA.

## 3. Experimental Section

### 3.1. Chemicals

All DNA samples were purchased from Integrated DNA Technologies (Coralville, IA, USA). The DNA sequences used in this experiment are: 12-mer, 5'-CACTGACCTGGG with a FAM (6-carboxyfluorescein) modification on the 5'- end; 12-mer cDNA, 5'-CCCAGGTCAGTG; 12-mer quencher, 5'-CCCAGGTCAGTG with Iowa Black FQ modification on the 3'- end; 24-mer, 5'-ACG CATCTGTGAAGAGAACCTGGG with a FAM modification on the 5'- end; 24-mer cDNA, 5'-TGC GTAGACACTTCTCTT; 44-mer, 5'-ACGCATCTGTGAAGAGAACCTGGGGGAGTATTGCGGAG GAAGGT; 44-mer cDNA, 5'-ACCTTCCTCCGCAATACTCCCCCAGGTTCTCTTCACAGATGC GT. Sodium chloride, magnesium chloride, 4-(2 hydroxyethyl)piperazine-1-ethanesulfonate (HEPES), and Tris(hydroxymethyl)aminomethane (Tris) were purchased from Mandel Scientific (Guelph, Ontario, Canada). Graphene oxide (GO) was synthesized using the modified Hummers method and was provided by our colleague Dr. Vivek Maheshwari. Millipore water was used for all the experiments.

### 3.2. Kinetics

The adsorption kinetics was monitored by a microplate reader (Infinite F200 Pro, Tecan, Switzerland) at 25 °C. 50 μL of samples were used for all the kinetic study. For DNA length comparison, 100 nM of FAM-DNA with 150 nM of cDNA in 100mM NaCl, 25mM HEPES buffer (pH 7.6) was heated at 85 °C and gradually cooled down to room temperature for hybridization. 40 μg/mL of GO was then added to the ssDNA or dsDNA samples in 100 mM NaCl, 25 mM HEPES buffer (pH 7.6) and the fluorescence signal was monitored for 2.5 h. For buffer condition comparison, 100 nM of 24-mer FAM-DNA was hybridized with 300 nM cDNA in 100 mM NaCl, 25 mM HEPES buffer (pH 7.6). 30 μg/mL of GO was then added to the DNA samples in either 100 mM NaCl, 25 mM HEPES buffer (pH 7.6) or 100 mM NaCl, 25 mM HEPES, 1 mM MgCl_2_ buffer (pH 7.6). The adsorption was monitored for 30 min.

### 3.3. Gel Electrophoresis

ssDNA/dsDNA mixtures with different ratio were first annealing in 100 mM NaCl 25 mM HEPES buffer (pH 7.6). The mixtures were then incubated with 100 μg/mL GO for 20 min in 100 mM NaCl, 25 mM HEPES buffer (pH 7.6). The samples were then centrifuged at 14,000 rpm and supernatants were collected and loaded into 15% non-denaturing polyacrylamide gel. Gel electrophoresis was running at 500 V for 1 h. The gel was illuminated with Invitrogen Safe Imager 2.0 Blue-Light Transilluminator (Life Technologies, Grand Island, NY, USA).

### 3.4. Melting Curves

Two different ratio of 12-mer dsDNA samples (1 μM FAM:500 nM quencher and 500 nM FAM:1 μM quencher) were hybridized in 100 mM NaCl, 25 mM HEPES buffer (pH 7.6). The duplex was first incubated with 20 μg/mL of GO at room temperature for 10 min. The mixture was then centrifuged at 14,000 rpm for 15 min and the supernatant was collected. The melting curve of the supernatant was recorded with CFX-96 real-time PCR (Bio-Rad, Mississauga, Canada).

## 4. Conclusions

In summary, we have compared the adsorption kinetics of ss- and ds-DNA as a function of DNA length, DNA/GO ratio, and salt concentration. Our data confirmed that both ss- and ds-DNA can be adsorbed by GO. In both cases, shorter DNAs were adsorbed more quickly than longer ones. This work has increased our understanding on DNA/GO interactions. Based on these studies, we further demonstrated that it was possible to selectively remove ss-DNA via GO adsorption. Since GO can be removed using a simple centrifugation step, a simple method for purifying ds-DNA was developed. To ensure complete removal of GO after the treatment, a pre-centrifugation step can be performed to eliminate small GO sheets that are more difficult to precipitate by centrifugation.
